# South Shields Guardians Withdraw Their Resolution

**Published:** 1914-02-07

**Authors:** 


### SOUTH SHIELDS GUARDIANS WITHDRAW THEIR RESOLUTION.

We are glad to note that the South Shields Guardians have withdrawn their embargo on the medical officer's requisition of alcohol, both the master and the clerk having followed The Hospital's lead in pointing out that the resolution in question was illegal. At a meeting of the Guardians last week the house committee's report contained the following:—The master reports: "With reference to the Board's decision that spirituous liquors are not to be further supplied to sick patients, that he is placed in a very invidious position owing to the incumbency upon himself to comply with any requisition which the medical officer may make for stimulants of this nature, and suggesting that in order to meet the difficulty, requisitions for any stimulants by the medical officer be complied with, and the amounts supplied from store be reported to each meeting of the hospital committee. The clerk points out that the resolution at the last Board meeting was, in effect, *ultra vires*, as the master is compelled to supply any stimulant which the medical officer considers necessary for the treatment of patients, and in view of this we have agreed with the master's suggestion." Thereupon, Mr. Appleton moved the adoption of the house committee's minutes including the above. Mr. Hall seconded, and we are glad to note that the resolution was carried without comment. It is often, as in this case, with the best intentions that Boards of Guardians attempt to trespass on the prerogative of their medical superintendent; and it is satisfactory that the South Shields Guardians have recognised their unwisdom so promptly.

As we remarked three weeks ago when noticing the passing of the resolution which has since been rescinded, Boards of Guardians not infrequently carry motions which, if not subsequently withdrawn, have to remain a dead letter. Such resolutions are generally foisted on the Board which adopts them by one or two members who wish to import their own private views on various public questions into the institutions for which they are responsible. That is what happened in this case, and we have already quoted the arguments and fallacious analogies (of which the controversy over alcohol contains so many) that were employed by the advocates of the resolution. No more useful function is exercised by a clerk to a Board of Guardians than that of mentor, which he so often has to be as to the legality of the various motions which come up for discussion at Board meetings. He plays the part of a useful breakwater between the private feelings of the Guardians and the powers and responsibilities with which the institutional officers are endowed.

It is in no invidious spirit of comparison that we boldly state that the medical officers of the Poor-Law infirmaries and the sick wards of the workhouses are the best guardians of the inmates and their welfare. It is inevitable that this should be so, since the medical officer has nothing else to think of save the efficiency of the institution under his control, while the Guardians are perforce engaged in the treacherous sea of municipal politics, and have to think of the ratepayers' attitude to any new departure as much as to the value of the departure itself. That is why the history of the Poor-Law infirmary has been a tenacious struggle on the part of the medical officers to raise the standard of equipment against often recalcitrant Guardians, who, unlike those who have been trained in the voluntary hospitals, often do not know how high that standard can be, and how direct is the right of the poorest classes of the population to benefit by it. Medical inspection is therefore universally welcomed by medical superintendents, who are only too glad to have an official backing for their pleas for improvement, and the regret is equally universal that such inspection is so infrequent and limited.

These being the facts it is essential that the powers given by the Orders of the Local Government Board to the medical officers of the institutions should be safeguarded, and we are glad to think that The Hospital has succeeded in securing prompt revision in the present case.

